# Programmed cell death in yeast by thionin-like peptide from *Capsicum annuum* fruits involving activation of caspases and extracellular H^+^ flux

**DOI:** 10.1042/BSR20180119

**Published:** 2018-04-27

**Authors:** Gabriel B. Taveira, Érica O. Mello, Sávio B. Souza, Renan M. Monteiro, Alessandro C. Ramos, André O. Carvalho, Rosana Rodrigues, Lev A. Okorokov, Valdirene M. Gomes

**Affiliations:** 1 Centro de Biociências e Biotecnologia, Universidade Estadual do Norte Fluminense Darcy Ribeiro, Campos dos Goytacazes 28013-602, RJ, Brazil; 2 Departamento de Farmácia, Universidade Iguaçu-Campus V, Itaperuna 28300-000, RJ, Brazil; 3 Centro de Ciências e Tecnologias Agropecuárias, Universidade Estadual do Norte Fluminense Darcy Ribeiro, Campos dos Goytacazes 28013-602, RJ, Brazil

**Keywords:** Antimicrobial peptides, apoptosis, Candida tropicalis, extracellular pH

## Abstract

*Ca*Thi is a thionin-like peptide isolated from fruits of *Capsicum annuum*, which has strong antimicrobial activity against bacteria, yeasts and filamentous fungi, and induced reactive oxygen species (ROS) in fungi. ROS are molecules that appear in the early stages of programmed cell death or apoptosis in fungi. Due to this fact, in this work we analyzed some events that may be related to process of apoptosis on yeast induced by *Ca*Thi. To investigate this possibility, we evaluated phosphatidylserine (PS) externalization, presence of active caspases and the ability of *Ca*Thi to bind to DNA in *Candida tropicalis* cells. Additionally, we investigated mitochondrial membrane potential, cell surface pH, and extracellular H^+^ fluxes in *C. tropicalis* cells after treatment with *Ca*Thi. Our results showed that *Ca*Thi induced PS externalization in the outer leaflet of the cell membrane, activation of caspases, and it had the ability for DNA binding and to dissipate mitochondrial membrane potential. In addition, the cell surface pH increased significantly when the *C. tropicalis* cells were exposed to *Ca*Thi which corroborates with ~96% inhibition on extracellular H^+^ efflux. Taking together, these data suggest that this peptide is capable of promoting an imbalance in pH homeostasis during yeast cell death playing a modulatory role in the H^+^ transport systems. In conclusion, our results strongly indicated that *Ca*Thi triggers apoptosis in *C. tropicalis* cells, involving a pH signaling mechanism.

## Introduction

Antimicrobial peptides (AMPs) are common molecules of the immune defense system in virtually all life forms, with representatives in organisms ranging from bacteria to plants and mammals [1–3]. They participate in an ancient defense system, innate immunity, which is the first line of defense for most living organisms during the early stages of an infection [4,5]. These peptides generally exhibit a wide range of inhibitory activity against viruses, bacteria, filamentous fungi, yeasts, protozoa, insects, and others [6–8].

AMPs have ~12–100 amino acid residues, molecular mass less than 10 kDa, and many positively charged lysine or arginine residues that confer a net positive charge at physiological pH [9–11]. The presence of a large number of cysteine residues (4, 6, or 8) that are connected in pairs forming disulphide bonds is an important characteristic for these peptides and gives them a high level of stability to extreme physicochemical conditions [12,13]. In general, AMPs present a mechanism of action that involves interaction with different membrane structures, which leads to the formation of pores and culminates in increased permeability, imbalance of ion homeostasis via inhibition on primary H^+^ transport systems and results in cell death [3,14–17]. However, the complete mechanism of action of AMPs remains unclear. Some reports have suggested that AMPs, such as the plant defensins, e.g. *Rs*-AFP_2_ from *Raphanus sativus* [18] and *Ap*Def_1_ from *Adenanthera pavonina* seeds [19], and other peptides, such as Papiliocin derived from the swallowtail butterfly *Papilio xuthus* larvae [20] and pleorocidin from *Pleuronectes americanus* (winter flounder) [21], can exert their antimicrobial activities through a process of programmed cell death or apoptosis. Apoptosis is a highly regulated cell death process that plays an essential role in the homeostasis and development of metazoans. It is essential to maintain cellular turnover by removing unwanted or damaged cells and to combat diseases or infections without an inflammatory reaction [22,23]. Many works have indicated that this process of programmed cell death is not exclusive to metazoans and can occur in unicellular organisms, such as yeast, which display typical apoptotic markers, such as reactive oxygen species (ROS) accumulation, phosphatidylserine (PS) externalization, DNA degradation, and chromatin condensation [19,20,24,25].

In a previous work, our group demonstrated that a thionin-like peptide, called *Ca*Thi, isolated from *Capsicum annuum* fruits, showed the endogenous production of ROS in *Candida tropicalis* amongst six *Candida* genera tested in an assay [26]. ROS was also observed in conidia of the fungus *Fusarium solani*, in which it was also shown that *Ca*Thi could induce the activation of caspases [27]. ROS are molecules that appear in the early stages of the programmed cell death process, and caspases play a central role in this process [28,29]. In this work, we originally addressed some events that might be related to the programmed cell death as part of the mechanism of action of *Ca*Thi in yeasts involving mainly the modulation of extracellular H^+^ flux, mitochondrial membrane potential, and activation of caspases.

## Materials and methods

### Biological material

*Capsicum annuum* L. fruits (accession UENF1381) were provided by the *Laboratório de Melhoramento Genético Vegetal, Centro de Ciências e Tecnologias Agropecuárias, Universidade Estadual do Norte Fluminense Darcy Ribeiro* (UENF), Campos dos Goytacazes, Rio de Janeiro, Brazil.

The yeasts *C. tropicalis* (CE017) and *Saccharomyces cerevisiae* (1038) were obtained from the *Departamento de Biologia, Universidade Federal do Ceará*, Fortaleza, Brazil. Yeasts were maintained on Sabouraud agar (1% peptone, 2% glucose, and 1.7% agar-agar) (Merck).

### Extraction and purification of *Ca*Thi

*Ca*Thi extraction and purification were accomplished as described by Taveira et al. [30].

### Detection of PS externalization

Spheroplasts of *S. cerevisiae* were obtained according to the method described by Okorokov and Lehle [31]. Briefly, the mid-logarithmic phase cells were transformed to spheroplasts by incubation at 37°C in buffer containing 1.2 M sorbitol, 10 mM Tris/HCl, pH 7.4, 30 mM β-mercaptoethanol, and 1 mg lyticase (Sigma)/1 g of wet cells. The spheroplasts (1 × 10^4^ spheroplasts.ml^−1^) were incubated in spheroplast buffer (1.2 M sorbitol, 10 mM Tris, pH 7.4) containing 10 μg.ml^−1^ of *Ca*Thi. The assay was performed on 96-well cell culture plates (Nunc) at 30°C for 2 h, with the final assay volume adjusted to 200 μl. After this time, the spheroplasts were washed in spheroplast buffer and centrifuged at 200 × ***g*** or 1280 rpm for 5 min. The pellet was resuspended in 100 μl Annexin-V-Fluo (Roche) marker kit, as described by the manufacturer, and incubated with constant agitation for 15 min, protected from light. Subsequently, the spheroplasts were analyzed by differential interference contrast (DIC) using an optical microscope equipped with a fluorescence filter to detect fluorescein (excitation wavelength: 450–490 nm, emission wavelength 500 nm). As a positive control, 2.5 mM of hydrogen peroxide was used under the respective conditions of *Ca*Thi, and a negative control was done by heating spheroplasts at 100°C for 1 min. The Annexin-V-Fluos Kit contains Annexin V, which is a calcium-dependent phospholipid-binding protein, which binds to negatively charged phospholipids with high specificity for PS, which is translocated to the external leaflet of the plasma membrane during the early stages of apoptosis. The kit also contains propidium iodide, a nucleic acid dye that is impermeable to living or apoptotic cells but stains dead cells with a red fluorescence and is thus used for the differentiation of necrotic cells.

### Caspase activity detection

Caspase activity detection was performed using the CaspACE FITC-VAD-FMK marker (Promega) as described by the manufacturer. The initial treatment conditions of *C. tropicalis* yeast cells with *Ca*Thi were done as described in the ‘Analysis of mitochondrial functionality’ section, except after incubation with *Ca*Thi, *C. tropicalis* cells were resuspended in Sabouraud medium, washed once in 500 μl PBS (10 mM NaH_2_PO_4_, 0.15 M NaCl) pH 7.4 and resuspended in 50 μl staining solution (supplied with the kit) containing 50 μM of the FITC-VAD-FMK marker. After 20 min of incubation at 30°C with constant orbital agitation at 500 rpm, the cells were washed in 500 μl PBS and resuspended in 20 μl PBS. Negative (incubated in the absence of *Ca*Thi) and positive (incubated with 300 mM acetic acid) control cells had the same treatment as did cells treated with the peptide. Cells were analyzed by DIC using an optical microscope equipped with a fluorescence filter to detect fluorescein (excitation wavelength: 450–490 nm, emission wavelength: 500 nm).

### Analysis of mitochondrial functionality

Mitochondrial functionality was assessed by the fluorescent dye Rhodamine 123 (Sigma). Rhodamine 123 is a cationic fluorescent dye that has high affinity with the electrical potential of membranes; thus, it marks active mitochondria in living cells, i.e. the loss of mitochondrial membrane potential is observed as a decrease in the fluorescent signal. Initially, *C. tropicalis* yeast cells (1 × 10^4^ cells.ml^−1^) were incubated in Sabouraud broth containing 10 μg.ml^−1^ *Ca*Thi, with the final assay volume adjusted to 200 μl. The assay was performed on 96-well cell culture plates (Nunc) at 30°C for 24 h. After incubation with *Ca*Thi, *C. tropicalis* cells were resuspended in the Saboraud medium and incubated with 10 μg.ml^−1^ Rhodamine 123, with constant orbital agitation at 500 rpm for 2 h and protected from light, and then analyzed by DIC under an optical microscope equipped with a fluorescence filter (excitation wavelength: 506 nm, emission wavelength: 530 nm). Control cells were treated in the same manner, except *Ca*Thi was excluded.

### Measurements of H^+^ flux using ion-selective vibrating probe system

*C. tropicalis* was grown in 40 ml Sabouraud broth for 24 h at 30°C and 250 rpm. After 24 h, an aliquot of 1 µl of cellular suspension of *C. tropicalis* was transferred to the center of a Petri dish (60 × 15 mm) containing 1 ml Sabouraud agar, and grown for 24 h at 30°C. After this growth period, 5 ml Sabouraud broth was gently added into the Petri dish, and in order to determine the extracellular voltage differences, proton flux, and cell surface pH, measurements of the H^+^ flux were performed by H^+^ selective vibrating probe.

H^+^-specific vibrating microelectrodes were manufactured as described by Feijó et al. [32] and Ramos et al. [33]. Micropipettes composed of 1.5 mm borosilicate glass capillaries were stretched and treated with dimethyl dichlorosilane (Sigma-Aldrich, U.K.). After silanization, the microelectrodes were back-loaded with electrolyte solution (15 mM KCl and 40 mM KH_2_PO_4_, pH 6.0 for H^+^, from 15 to 20 mm microelectrode column) and then front-loaded with respective ion-selective liquid exchange cocktail (Fluka, from 20 to 25 µm electrolyte column). An Ag/AgCl wire electrode holder (World Precision Instruments) was inserted into the back of the microelectrode for electrical contact with the bathing solution. The ground electrode was used as a dry reference (DRIREF-2, World Precision Instruments) and was inserted into the sample bath. The microelectrodes were calibrated by measuring the background signal at the beginning and end of each experiment using standard solutions covering the experimental range of each ion. Both the slope and intercept of the calibration line were used to calculate the respective ion concentration from the mV values measured during the experiments.

The treatment with *Ca*Thi was performed in *C. tropicalis* cells after determination of each H^+^ flux at each colony (assay was done twice and in triplicate, *n*=6). Data acquisition was stopped and the respective treatments were added in the Petri dishes and the H^+^ flux was measured for a minimum of 5 min or until they reached the steady state. After that, a background reference was taken and H^+^ flux was recorded again.

### Analysis of DNA-binding capability of *Ca*Thi

DNA from *C. tropicalis* was extracted using the DNeasy plant mini kit (Qiagen) from a culture of *C. tropicalis* grown in Sabouraud broth for 24 h. The extracted DNA was quantitated using NanoDrop 2000 (Applied Biosystems). The test is based on the alteration of electrophoretic mobility due to the binding of DNA to protein. This binding results in the formation of a complex resulting in a different electrophoretic mobility than free DNA.

The assay was performed as described by Park et al. [34]. Briefly, a 0.8% agarose gel was prepared according to Sambrook and Russel [35]. One hundred nanograms of DNA from *C. tropicalis* was incubated with 10 µg.ml^−1^ of *Ca*Thi plus 20 µl binding buffer (5% glycerol, 10 mM Tris/HCl pH 8.0, 1 mM EDTA, 1 mM DTT, 20 mM KCl, and 50 µg.ml^−1^ BSA), and the mixture was incubated for 30 min at 30°C. Water was used as a negative control, and 10 µg.ml^−1^ of poly-l-lysine (Sigma), which has the ability to bind to DNA, served as a positive control. Gel images were captured using an ImageQuant LAS 500 (GE Healthcare).

## Results

### PS externalization in *S. cerevisiae* yeast cell by *Ca*Thi

**Figure 1:**
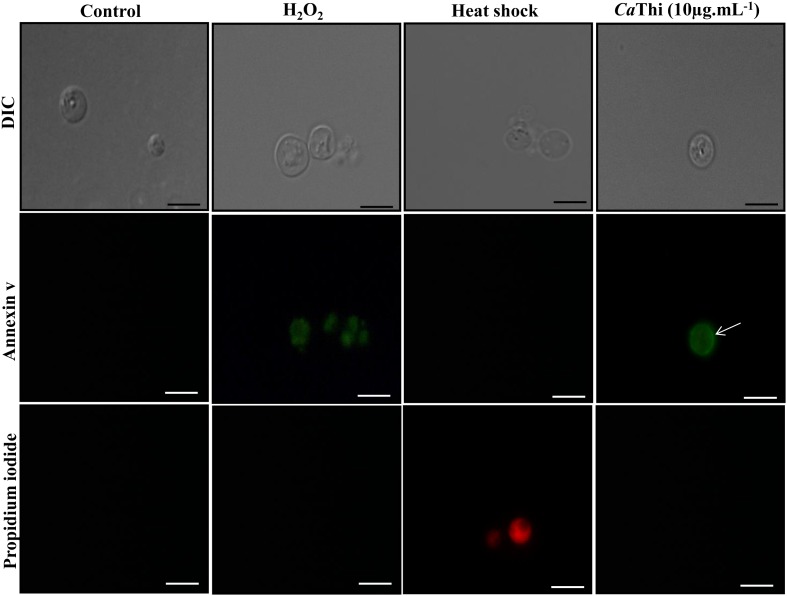
*S. cerevisiae* spheroplasts incubated for 2 h with 10 μg.ml^−1^ *Ca*Thi and then treated with Annexin V (AnnV) and propidium iodide (PI), showing PS exposure to the outer monolayer (green fluorescence) (open arrow) Control (spheroplasts in assay medium) are AnnV^−^ and PI^−^, H_2_O_2_ (H_2_O_2_ treated spheroplasts) are AnnV^+^ and PI^−^, heat shocked cells (spheroplasts treated at 100°C) are AnnV^−^ and PI^+^ (red fluorescence). Bars 5 μm.

Positive labeling for PS induced by *Ca*Thi was observed, visualized by the green fluorescent contour on the spheroplast of *S. cerevisiae*, suggesting that the translocation of PS from the internal leaflet to the external leaflet of the plasma membrane occurred. This result indicates that *Ca*Thi may cause an apoptotic effect on *S. cerevisiae* cells, as observed by PS exposure. A similar effect was observed in the positive control, where the cells were incubated with hydrogen peroxide. However, in the negative control (heated), propidium iodide labeling was observed, indicating necrotic death (Figure 1).

### Caspase activity detection

**Figure 2:**
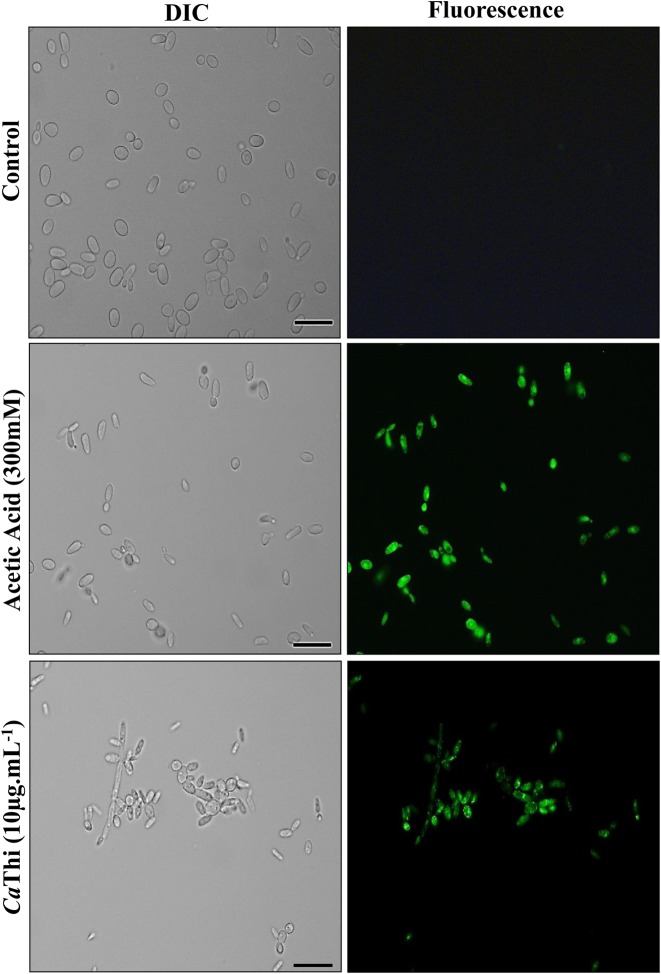
Activity of caspase in *C. tropicalis* cells after 24 h of incubation with 10 μg.ml^−1^ of *Ca*Thi Control cells and cells treated with *Ca*Thi were incubated with the FITC-VAD-FMK probe and analyzed by fluorescence microscopy. Green fluorescence indicates positive staining for caspase activity. Bars = 10 μm.

The detection of caspase activity was performed using the CaspACE FITC-VAD-FMK *in situ* marker. The FITC-VAD-FMK marker is an analog of the caspase inhibitor Z-VAD-FMK (carbobenzoxy-valyl-alanyl- aspartyl-[O-methyl] fluoromethylketone). The N-terminus carbobenzoxy (Z) is replaced by FITC, creating a fluorescent label for apoptosis. This complex enters the cell, where it acts as a pseudosubstrate that irreversibly inhibits caspases by binding to the cysteine residue at its active site and becomes fluorescent. To verify the occurrence of apoptotic events induced by *Ca*Thi in *C. tropicalis* cells, the activity of caspase type enzymes was investigated. Our results show an induction of caspase activity, suggesting a programmed cell death occurring by an apoptotic pathway (Figure 2).

### Analysis of mitochondrial functionality in cells of *C. tropicalis*

**Figure 3:**
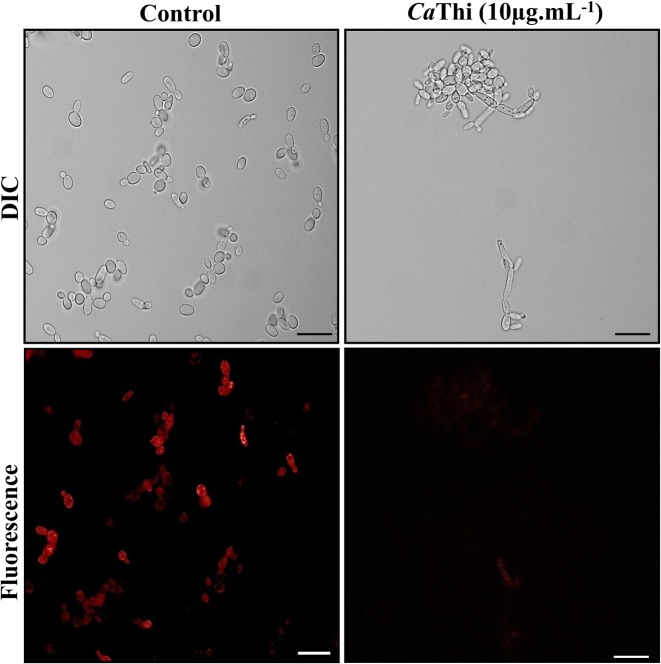
Cells of *C. tropicalis* after mitochondrial functionality assay, visualized by fluorescence microscopy using Rhodamine 123 fluorescent probe Cells were treated with 10 μg.ml^−1^ of *Ca*Thi for 24 h and then analyzed for mitochondrial functionality. Control cells were treated only with Rhodamine 123 probe. Bars = 10 μm.

The mitochondrial functionality assay was evaluated through the fluorescent dye Rhodamine 123. *C. tropicalis* cells, after 24 h of treatment with 10 μg.ml^−1^ *Ca*Thi, presented with diminished mitochondrial signals, as observed by the weak fluorescence of the dye Rhodamine 123. Control cells with functional mitochondria evinced a strong signal of Rhodamine 123 fluorescence (Figure 3). This result indicates that after treatment with *Ca*Thi, cells of *C. tropicalis* lost their electrical membrane potential, causing the dysfunction of their mitochondria.

### Analysis of H^+^ flux using an ion-selective vibrating probe

**Figure 4:**
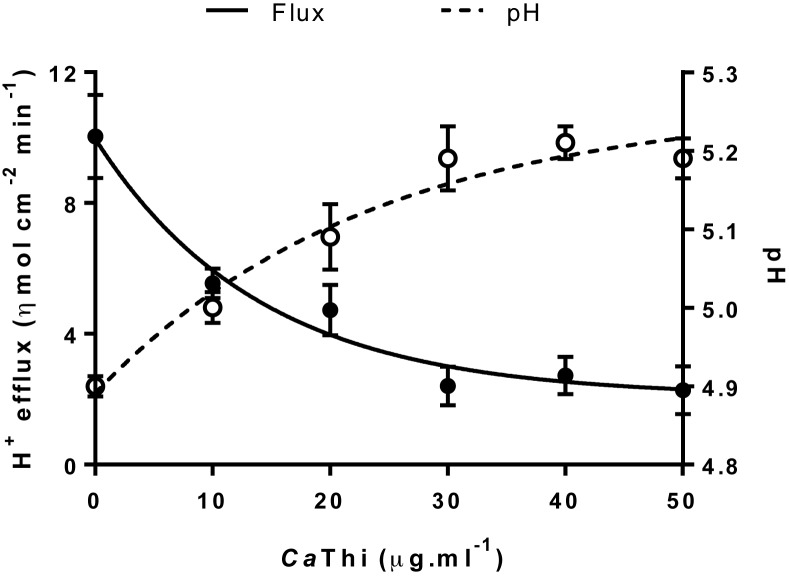
Dose–response curve of the effects of *Ca*Thi peptide on extracellular pH and H^+^ efflux in the *C. tropicalis* yeast The graphic was done with the mean and S.D. of one independent assay.

**Figure 5:**
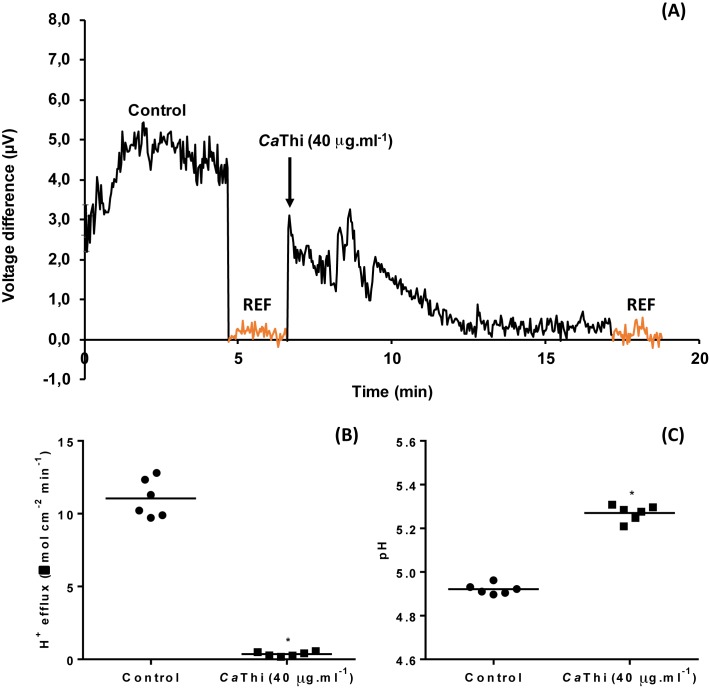
Dose–response curve of the effects of *Ca*Thi peptide on extracellular voltage difference (A), proton (H^+^) fluxes (B), and cell surface pH (C) in the *C. tropicalis* yeast For H^+^ effluxes and pH data, means are significantly different by Student *t* test at *P*≤0.01 indicated by ‘*’. REF represents the background reference.

Extracellular voltage difference, proton fluxes, and cell surface pH were measured in *C. tropicalis* yeast using an H^+^-selective vibrating probe. The dose–response test showed a significant inhibition of the H^+^ efflux in the yeast cells after treatment with *Ca*Thi (Figure 4). An inverse behavior was observed with the extracellular pH (Figure 4). A stable H^+^ voltage difference was recorded in the presence or absence of the *Ca*Thi peptide (Figure 5A). Before exposure to 40 µg.ml^−1^ *Ca*Thi, the yeast cells showed a steady state of extracellular H^+^ efflux activity of 11.04 ± 1.39 ηmol.cm^−2^.min^−1^, but after the peptide treatment, the H^+^ efflux had effluxes of ~0.36 ± 0.15 ηmol.cm^−2^.min^−1^ (*P*≤0.0001, *t*test); i.e. they presented a significant inhibition of 96.84% (Figure 5B). Consequently, the cell surface pH had a significant increase when the yeast cells were exposed to *Ca*Thi (Figure 5C). However, after 10 min of removal of *Ca*Thi from the medium, the basal H^+^ efflux and cell surface pH returned to the normal level, suggesting that this inhibition is *Ca*Thi dependent (data not shown).

### DNA electrophoretic mobility assay

**Figure 6:**
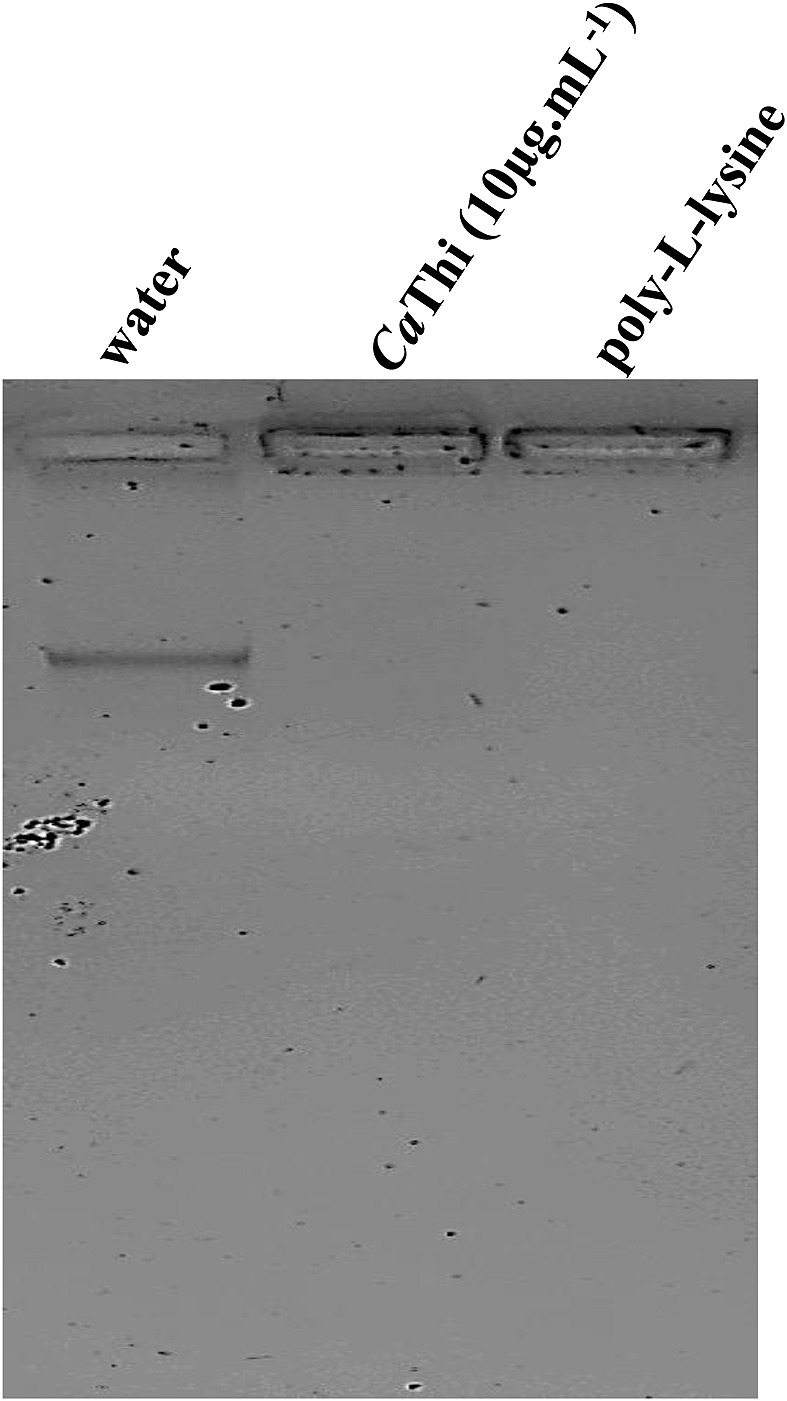
Dose–response curve of the effects of *Ca*Thi peptide on extracellular pH and H^+^ efflux in the *C. tropicalis* yeast First line, control, mobility of 100 ng of DNA and binding buffer. Second line, mobility of 100 ng DNA incubated with 10 μg.ml^−1^ of *Ca*Thi. Third line, positive control, mobility of 100 ng of DNA incubated with 10 μg.ml^−1^ of poly-l-lysine.

In a previous work, we demonstrated that *Ca*Thi is able to internalize and suggested a possible target in the nucleus of *C. tropicalis* cells [26]. Thus, we hypothesized that *Ca*Thi could interact with the DNA of these cells. Therefore, we looked for evidence of this occurrence by verifying a possible change in DNA mobility. Our result indicates that *Ca*Thi at 10 μg.ml^−1^ was able to bind to the DNA molecule and that this binding results in a complex that has a different electrophoretic mobility from the free DNA, preventing its entry into the agarose gel mesh, as may be observed in the test (second lane) and in the positive control when using poly-l-lysine (third lane). This result was corroborated when we visualized the negative control only in the presence of water, where the normal electrophoretic mobility of the DNA molecule is observed (first lane) (Figure 6).

## Discussion

*Ca*Thi causes viability loss in yeast cells (*C. tropicalis*) and in filamentous fungi (*F. solani*) and increased ROS production in both fungi [26,27]. Importantly, ROS are the main inducers of apoptosis in fungi [36]. Taken together, this information led us to hypothesize that *Ca*Thi triggers an apoptosis pathway in treated fungi. Mitochondria play a crucial role in cell survival by generating ATP and controlling apoptosis, the cell cycle and other essential metabolisms. Mitochondria generate ATP through oxidative phosphorylation during aerobic respiration, where glucose, pyruvate, and NADH molecules are oxidized, generating ROS as metabolic byproducts [37–39]. Therefore, mitochondria and ROS play a major role in the induction of apoptosis under normal physiological conditions. Apoptosis is characterized by noticeable biochemical and physical alterations that occur in the cytoplasm and in cell components such as the nucleus and plasma membrane. These alterations occur in an ordered time frame and are generated by two main signaling pathways, the intrinsic or mitochondrial and the extrinsic pathways; both pathways start with the activation of caspases [40,41]. To investigate the possibility of the induction of apoptosis by *Ca*Thi in yeast cells, some of the altered characteristics were evaluated as PS externalization, the presence of active caspases, and the dissipation of mitochondrial membrane potential. Additionally, we investigated the H^+^ flow in *C. tropicalis* cells *in vivo* and the binding of *Ca*Thi to DNA isolated from the same yeast.

To verify the PS externalization, we used the protoplasts of *S. cerevisiae* as a model. We used this model because the technique of obtaining spheroplasts was already standardized in our group for *S. cerevisiae* and because of the requirement that the cell wall be absent for the experimental detection of PS externalization. The translocation of PS from the inner leaflet to the outer leaflet of the plasma membrane occurs during the early stages of apoptosis, and this externalization can be detected by the labeling with Annexin V, which binds to phospholipids with negative charges but with high specificity for PS [20]. When our protoplasts were analyzed by fluorescence microscopy, we observed only Annexin V labeling (Figure 1), suggesting that *Ca*Thi induces PS externalization. This result indicates that a programmed cell death pathway may have been activated in this yeast. Similarly, other AMPs isolated from different sources are capable of inducing apoptosis in yeast cells. Hwang et al. [20] found that papiliocin peptide, isolated from *P. xuthus*, induced the accumulation of ROS and free radicals that are important regulators of apoptosis in *C. albicans*. These cells treated with papiliocin showed a series of changes normally seen in cells undergoing early apoptosis, such as the translocation of PS from the inner side to the outer side of the plasma membrane. Lee et al. [42] demonstrated by Annexin-FITC labeling and the TUNEL assay that coprisin peptide, similar to defensin, isolated from *Copris tripartitus* (beetle), was involved in both the early and late stages of apoptosis, increasing the intracellular level of ROS and free hydroxyl radicals in *C. albicans* cells.

After verifying the PS translocation event in the presence of *Ca*Thi, we verified the presence of activated caspases and the dissipation of mitochondrial membrane potential by rhodamine 123 [43–45]. Caspases are specific aspartate proteases containing cysteines, are typically activated in the early stages of apoptosis and play a central role in apoptotic signaling network [29]. In these two tests, we verified that *Ca*Thi increases the level of caspase activity in *C. tropicalis* cells and could cause the collapse of the mitochondrial membrane potential at the same cells (Figures 2 and 3). The papiliocin AMP also showed these activities, causing the dissipation of mitochondrial membrane potential and the activation of caspases. Some late events of apoptosis have also been observed, such as the fragmentation and condensation of DNA. These data suggest that papiliocin led *C. albicans* cells to apoptosis via ROS accumulation [20]. Another AMP called psacotheasin, a knotin family peptide, isolated from larvae of the beetle *Psacothea hilaris*, was able to provoke various apoptotic effects in *C. albicans*, such as PS externalization, mitochondrial membrane depolarization, and increased activity of caspase, in addition to DNA fragmentation and condensation [46]. In 2011, Cho and Lee [47] suggested that areninin-1 from *Arenicola marina* (lugworm), has antifungal activity by inducing apoptosis in *C. albicans*, which caused an increased production of intracellular ROS, mitochondrial membrane depolarization, caspases activation, plasma membrane depolarization, and translocation of PS to the external leaflet of membrane surfaces. This same yeast showed morphological changes in the nucleus and structural changes in the DNA molecule. In conjunction with the results already demonstrated for other AMPs, our data suggest that *Ca*Thi possesses antifungal activity by activation of the apoptosis pathway.

The H^+^ fluxes through the plasma membrane exert essential functions in the physiology of the fungal cell. It is normally mediated by the plasma membrane H^+^-ATPase and interference in this flux can lead to cell death. H^+^ flux was monitored by measuring the H^+^ flux in *C. tropicalis* cells. The results presented here showed that after treatment with *Ca*Thi, the H^+^ efflux had a significant inhibition of ~96%, thus clearly demonstrating that this peptide is capable of causing an imbalance in H^+^ homeostasis (Figures 4 and 5). Diz et al. [48] and Ribeiro et al. [49] demonstrated that peptides present in a fraction obtained from seeds of *Capsicum annuum* also inhibited 100% of the H^+^ efflux in *S. cerevisiae* at a concentration of 160 μg.ml^−1^. In contrast, an LTP extracted from coffee (*Coffea canephora*) called *Cc*-LTP_1_ increased the acidification of the medium, i.e. an increase in the H^+^ efflux, in cells of *S. cerevisiae, C. albicans*, and *C. tropicalis* [50]. Therefore, apoptotic induction may alter the ionic gradient across the plasma membrane, leading to an ionic imbalance and the depolarization of this membrane [21,47,51]. Andrés et al. [17] found that a human lactoferrin (hLf), a protein of the innate immune system, affects the activity of mitochondrial and plasma membrane H^+^-ATPase, inducing an apoptosis-like process in yeast. Indeed, changes in membrane potential and transmembrane ion fluxes are amongst the fastest responses of the cell to external stimuli [52,53]. The H^+^ efflux in yeast cells are linked to the activity of plasma membrane H^+^-ATPase, which plays a crucial role for cell growth, providing energy for nutrient uptake by channels and secondary transporters [54,55]. The formation of a proton electrochemical gradient is necessary for the maintenance of the intracellular pH and cell survival. Thus, the monitoring of the proton fluxes in yeasts could be a new tool to select innovative and more effective antifungal compounds [55].

Thionins may be a new group of DNA-binding proteins, as reported by molecular modeling studies, suggesting that they may interact with DNA within the microorganism cells [56,57]. In a previous study, Taveira et al. [26] demonstrated that *Ca*Thi is internalized in *C. tropicalis* cells and is localized in the nucleus of these cells. An obvious nuclear target should be DNA, and to verify if *Ca*Thi has this binding ability, we performed a *Ca*Thi binding assay on DNA. The results demonstrated that *Ca*Thi binds to DNA of *C. tropicalis*, thus reinforcing our hypothesis of a possible nuclear target (Figure 6). One possibility for microbial inhibition based on DNA binding capability is the blockage of molecular synthesis, as demonstrated by the AMPs PR-39 (from pig) and cryptdin-2 (a Paneth cell defensin from mouse) [58,59]. In general, AMPs are multifunctional molecules as observed by the effects they induced after interaction with microorganism cells and, thus, they are supposed to have many cellular targets which altogether led to microorganism death [60]. Moreover, the effects of PS externalization, caspase activation, mitochondrial membrane depolarization, and H^+^ efflux inhibition which suggest an apoptotic cell death, may be summed to the effect to the DNA binding leading to cell death. Thus, we can assume that directly binding to DNA through electrostatic interactions, as peptides have a positive overall charge while DNA molecule has a negative charge, *Ca*Thi may have promoted changes in the cellular DNA structure, leading to cell death [47]. However, whether these *in vitro* observations have significance *in vivo* is not known, and more studies will be needed for this direct connection to be fully established.

Herein, we further increase the understanding of the action mechanism of fungal inhibition by *Ca*Thi. The data presented in the present study suggest a pH signaling mechanism, in which *Ca*Thi triggers cell death in *C. tropicalis* based on the key markers of yeast apoptosis, such as PS externalization, presence of active caspase, and dissipation of mitochondrial membrane potential. Additionally, we originally showed that *Ca*Thi regulates the external pH of the cell, acting directly on the extracellular H^+^ fluxes, which could be an important target to identify if any compound has antifungal function.

## Abbreviations

AMP: antimicrobial peptide
DIC: differential interference contrast
PS: phosphatidylserine
ROS: reactive oxygen species
